# Genetic diversity and antimicrobial resistance among isolates of *Escherichia coli* O157: H7 from feces and hides of super-shedders and low-shedding pen-mates in two commercial beef feedlots

**DOI:** 10.1186/1746-6148-8-178

**Published:** 2012-09-26

**Authors:** Kim Stanford, Chelsey A Agopsowicz, Tim A McAllister

**Affiliations:** 1 Alberta Agriculture and Rural Development, Agriculture Centre, 100-5401 1st Ave, S, Lethbridge, AB, T1J 4 V6, Canada; 2 Agriculture and Agri-Food Canada, Lethbridge Research Centre, Lethbridge, AB, T1J 4B, Canada

**Keywords:** Super-shedder, *E. coli* O157:H7, Cattle, PFGE

## Abstract

**Background:**

Cattle shedding at least 10^4^ CFU *Escherichia coli* O157:H7/g feces are described as super-shedders and have been shown to increase transmission of *E. coli* O157:H7 to other cattle in feedlots. This study investigated relationships among fecal isolates from super-shedders (n = 162), perineal hide swab isolates (PS) from super-shedders (n = 137) and fecal isolates from low-shedder (< 10^4^ CFU/g feces) pen-mates (n = 496) using pulsed-field gel electrophoresis (PFGE). A subsample of these fecal isolates (n = 474) was tested for antimicrobial resistance. Isolates of *E. coli* O157:H7 were obtained from cattle in pens (avg. 181 head) at 2 commercial feedlots in southern Alberta with each steer sampled at entry to the feedlot and prior to slaughter.

**Results:**

Only 1 steer maintained super-shedder status at both samplings, although approximately 30% of super-shedders in sampling 1 had low-shedder status at sampling 2. A total of 85 restriction endonuclease digestion clusters (REPC; 90% or greater similarity) and 86 unique isolates (< 90% similarity) were detected, with the predominant REPC (30% of isolates) being isolated from cattle in all feedlot pens, although it was not associated with shedding status (super- or low-shedder; *P* = 0.94). Only 2/21 super-shedders had fecal isolates in the same REPC at both samplings. Fecal and PS isolates from individual super-shedders generally belonged to different REPCs, although fecal isolates of *E. coli* O157:H7 from super- and low-shedders showed greater similarity (*P* < 0.001) than those from PS. For 77% of super-shedders, PFGE profiles of super-shedder fecal and PS isolates were distinct from all low-shedder fecal isolates collected in the same pen. A low level of antimicrobial resistance (3.7%) was detected and prevalence of antimicrobial resistance did not differ among super- and low-shedder isolates (*P =* 0.69), although all super-shedder isolates with antimicrobial resistance (n = 3) were resistant to multiple antimicrobials.

**Conclusions:**

Super-shedders did not have increased antimicrobial resistance compared to low-shedder pen mates. Our data demonstrated that PFGE profiles of individual super-shedders varied over time and that only 1/162 steers remained a super-shedder at 2 samplings. In these two commercial feedlots, PFGE subtypes of *E. coli* O157:H7 from fecal isolates of super- and low-shedders were frequently different as were subtypes of fecal and perineal hide isolates from super-shedders.

## Background

Cattle shedding at least 10^4^ CFU/g *E. coli* O157 in feces were first termed “super-shedders” by Matthews et al. [1], and the role of super-shedders in contamination of the food supply has been much investigated. As duration of super-shedding is unknown, Carlson et al. [2] proposed that persistent low-level shedders of *E. coli* O157:H7 were a greater food safety risk than were intermittent super-shedders. In contrast, 47% [3] to > 90% [4] of the shedding of *E. coli* O157:H7 within feedlot pens has been attributed to super-shedders, even though these animals are thought to represent < 10% of the cattle population [4,5]. As most previous studies of super-shedders have evaluated a single point in time, it is also possible that many cattle are super-shedders for only a brief period. Accordingly, Robinson et al. [6] reported that within-animal variation of shedding *E. coli* O157 was greater than that among animals over time.

As the majority of cattle positive for *E. coli* O157:H7 shed < 100 CFU/g of feces [7], a super-shedder releasing up to 10^9^ CFU/g feces [3] even for short durations could represent a significant point source of environmental and possibly food contamination. In pens of 8 cattle, Cobbold et al. [8] observed that isolates of *E. coli* O157 were similar among super-shedders and pen mates based on pulsed-field gel electrophoresis (PFGE) analyses, while Stanford et al. [9] demonstrated that contaminating the perineum of a steer with feces containing 10^6^ CFU *E. coli* O157:H7 resulted in 7 of 8 steers in the pen acquiring and shedding this organism. In larger groups, presence of a super-shedder in a load of 20 to 50 cattle during shipment to slaughter has increased carcass contamination with *E. coli* O157 [10,11]. However, it is not known if a similar level of transmission from super-shedders to pen mates occurs in commercial feedlot pens where 100 to 200 individuals may be housed.

Previously we reported that super-shedders increased the incidence of perineal swab (PS) contamination with *E. coli* O157 in pens of commercial cattle [3]. The objectives of the present study were to determine genetic relationships among subtypes of *E. coli* O157:H7: 1) shed in feces by super- and low-shedders (< 10^4^ CFU/g feces) in commercial feedlot pens; 2) shed in feces and detected on PS of super-shedders. Pulsed-field gel electrophoresis was used to characterize super-shedder subtypes of *E. coli* O157:H7 as it has been used to evaluate genetic relationships and the transmission of *E. coli* O157 throughout the beef supply chain [12-14]. As antibiotic treatment can select for resistant bacteria and mutator alleles leading to increased virulence [15] and isolates from super-shedders may also have heightened virulence, antimicrobial resistance (AMR) profiles were also compared in isolates of *E. coli* O157:H7 from super- and low-shedder pen mates.

## Results and discussion

### Occurrence of super-shedders

Although 153 super-shedders were detected in sampling 1 (Table 1), only 1 steer was a super-shedder at both sampling dates. The number of super- and low- shedders markedly declined at the second sampling, likely due to the prevalence of *E. coli* O157 generally declining seasonally from summer to fall [7,16] and after a month of acclimation of cattle within a feedlot [17] However, the proportion of super- as compared to low-shedders also declined at the second sampling, a result that may reflect the transition of super-shedders to low-shedder status as approximately 30% of low-shedders in the second sampling were super-shedders in sampling 1. Of the 21 super-shedders which were low-shedders in sampling 2, only 2 steers had fecal isolates of *E. coli* O157:H7 in the same REPC at both samplings. Transition in PFGE subtypes within super-shedders was likely due to both transmission of *E. coli* O157:H7 among animals and mutation events. Transmission was likely for 3 steers that had isolates within a prevalent REPC at the second sampling and isolates from 3 different REPC specific to super-shedders in sampling 1. Mutation or transfer of *E. coli* O157:H7 from other sources was likely for 5 steers where isolates in the second sampling were from REPC that were specific to sampling 2 and a single pen within a feedlot.

**Table 1:** **Pens of cattle sampled in Alberta Canada in 2007 with 1 fecal grab sample and 1 hide swab collected per steer at each sampling and PFGE performed on samples positive for *E. coli *O157:H7**

**Feedlot**	**A**							**B**				**Total**
Pen	1	2	3	4	5	6	7	1	2	3	4	
cattle (n)	153	152	153	253	258	155	233	151	235	138	106	1987
Sample 1 date	May 22	Jun 4	Jul 20	Jul 27	Sep 26	Oct 9	Oct 23	Jul 6	Jul 12	Aug 10	Aug 30	
SS fecal isolates (n)	2	36	12	19	7	3	0	19^x^	44	10	1	153
SS perineal swabs (n)	2	30	11	17	3	3	0	17	40	7	1	131
LS fecal isolates (n)	13	37	100	54	24	2	1	36	107	20	31	425
Sample 2 date	Sep 24	Sep 6	Oct 3	Oct 17	Dec 10	Nov 29	Dec 6	Sep 28	Sep 28	Nov 14	Nov 27	
SS fecal isolates (n)	1	0	0	0	0	0	0	8^x^	0	0	0	9
SS perineal swabs (n)	0	0	0	0	0	0	0	6	0	0	0	6
LS fecal isolates (n)	3	10	0	2	3	0	1	40	9	3	0	71
LS fecal isolates from former SS (n)	0	1	0	2	2	0	0	7	9	0	0	21
Total isolates tested by PFGE (n)	21	113	123	92	37	8	2	126	200	40	33	795
Total REPC (n)	1	12	14	14	6	1	1	20	13	6	6	85^y^
Total unique isolates (n)	8	33	11	14	12	6	0	13	17	8	11	86^y^
SS fecal & SS perineal swab in same REPC (n)	1	1	0	0	0	0	NA^z^	0	0	0	0	2

Numbers of super–shedders (SS)^v^ and low- shedders (LS)^w^ within pens. Numbers of, restriction endonuclease digestion clusters (REPC, 90% or greater similarity) and unique isolates (< 90% similarity) within and across pens.

^v^Super-shedder, shedding at least 10^4^ CFU E. coli O157:H7/g feces.

^w^Low-shedder, shedding < 10^4^ CFU E. coli O157:H7/g feces.

^x^One steer was a super-shedder at both samplings.

^y^Total comparing all isolates across pens.

^z^NA, not applicable, no super-shedders in pen.

As only one steer was a super-shedder at both samplings, our results support the contention of Robinson et al. [6] that individual cattle are super-shedders for short periods and that levels of shedding vary widely among sampling time points. However, as former super-shedders constituted a large proportion of the low-shedder individuals identified at the second sampling (i.e., 30%), high-levels of shedding may also be associated with greater persistence of *E. coli* O157 within the gastro-intestinal tract [4,8,18], although results of the current study demonstrate transition of fecal PFGE subtypes by super-shedders over time (Additional file 1, Additional file 2). With only 2 samples collected per steer at least 1 month apart, estimates of the duration of high-level shedding by super-shedders were not possible in this study, a factor that will be investigated in future studies through a more frequent sampling routine. Robinson et al. [6] used an intensive regime which involved sampling individuals every 3 h, a protocol that would be almost impossible to implement under commercial feedlot conditions.

### Pulsed-field gel electrophoresis – most common profile

A level of 90% similarity (< 3 band differences) was chosen to define REPCs as PFGE profiles differing by one or two bands are considered to be highly related [19]. The REPC most frequently detected (REPC A) was also the only PFGE profile that was present in all pens at both feedlots (Table 2), likely representing an *E. coli* O157:H7 subtype that is common to feedlots within the sampling area. In sampling 1, 28% of isolates belonged to REPC A, with 34% of isolates in REPC A at sampling 2. Within pens, membership in REPC A was not homogeneous and in 2 pens this PFGE profile was confined to low-shedders, while in 4 pens this PFGE profile was found only in super-shedders. As REPC A was not related to *E. coli* O157:H7 shedding status of the cattle (*P =* 0.94; Table 3), the abundance of isolates from this REPC is likely indicative of the fitness of this *E. coli* O157:H7 in the gastrointestinal tract or feedlot environment. Accordingly, Carlson et al. [2] determined that persistence and dominance of *E. coli* O157:H7 strains isolated from feedlot cattle was influenced by genotype and possibly related to the degree of adherence to intestinal epithelial cells

**Table 2:** **Numbers of super-shedder (SS)**^**v **^**and low-shedder (LS)**^**w **^**isolates from both samplings sharing restriction endonuclease digestion pattern clusters (REPC, 90% or greater similarity) and description of most prevalent REPC, REPC A by feedlot and pen**

		**All REPC**^**x**^				**REPC A only**			
**Feedlot**	**Pen**	**No.SS**	**No.LS**	**No. SS sharing REPC with LS (%)**	**No. SS in SS only REPC (%)**	**No. isolates**	**No, SS fecal**	**No. SS hide**	**No. LS fecal**
A	1*	3	16	1 (33)	2 (67)	15	1	1	13
	2*	36	47	12 (33)	24(67)	41	12	6	23^y^
	3	12	100	11(92)	1(8)	5	4	1	0
	4	19	56	0 (0)	19 (100)	11	9	2	0
	5	7	27	0(0)	7 (100)	4	4	0	0
	6	3	2	0(0)	3(100)	1	1	0	0
	7	0	1	NA^z^	NA	2	0	0	2
B	1	27	76	4(15)	23 (85)	12	12^y^	0	0
	2*	44	116	9 (20)	35 (80)	120	9	10	101^y^
	3*	10	23	1(10)	9 (90)	19	1	0	18
	4	1	31	0 (0)	1(100)	5	0	0	5
Total		162	496	38 (23)	124 (77)	235	53	20	162

Pens where SS and LS isolates belonged to REPC A are marked by *, percentages are bracketed.

^v^Super-shedder, shedding at least 10^4^ CFU E. coli O157:H7/g feces.

^w^Low-shedder, shedding < 10^4^ CFU E. coli O157:H7/g feces.

^x^All REPC, number of super-shedders based on fecal isolates, super-shedder fecal and hide swab isolates included in REPC shared with low-shedders.

^y^Includes isolates from the second sampling.

^z^NA, not applicable, no super-shedders in pen.

**Table 3:** **Relationships among super-shedder status (yes/no), sampling (1 vs 2) and membership in the most prevalent restriction endonuclease digestion pattern cluster, REPC A , as compared to all other REPC for fecal isolates of *E. coli *O157:H7 collected in Alberta Canada in 2007 from generalized linear mixed model analysis**^**z **^

**Variable**	**Odds Ratio**	**95% C.I.**	**Significance**
Sampling	1.27	0.68 – 4.37	*P* = 0.18
Super-shedder	0.98	0.64 – 1.51	*P* = 0.94

^z^Pen as random effect with covariance parameter estimate = 1.48 and standard error = 0.83.

As REPC A was of similar prevalence in both sampling 1 and 2 (*P* = 0.18; Table 3), there was no evidence of a transition over time to a single dominant subtype of *E. coli* O157 which would account for the majority of isolates, likely due to the wide diversity of PFGE subtypes in calves entering the feedlot [14]. Considering all PFGE subtypes, genetic diversity tended to increase slightly from sampling 1 (average of 4 isolates per subtype) to sampling 2 (average of 3 isolates per subtype), although any change in diversity was mostly likely a reflection of the disparity in numbers of isolates of *E. coli* O157:H7 collected (709 isolates in sampling 1 as compared to 86 in sampling 2).

### Comparison of super-shedder hide swab and fecal grab isolates

Due to the volume of isolates collected and the laborious nature of PFGE, only one isolate was analysed per sample type which may have under-estimated the genetic diversity of *E. coli* O157:H7 compared to analysis of multiple isolates [20], although analysis of a single isolate is generally indicative of the dominant strain [21,22]. Consequently, the lack of congruity between simultaneously-collected fecal and PS isolates from individual super-shedders was surprising, as only 2 super-shedder steers had PS and fecal isolates in the same REPC (Table 1).

Most previous studies have used PFGE to characterize either hide or fecal isolates of *E. coli* O157:H7 [14,22-24]. Avery et al. [12] found some commonality of PFGE subtypes from *E. coli* O157:H7 isolated from the hide and feces of the same animal, but only sampled 5 cattle. Arthur et al. [25] found similarities as 9 hide and 22 fecal PFGE profiles for *E. coli* O157:H7 from a single feedlot belonged to only 2 REPC. In contrast, from 795 *E. coli* O157:H7 isolates in the present study, a total of 85 REPCs and 86 unique isolates were detected (Table 1). Genetic diversity of *E. coli* O157:H7 varies widely by location, ranging from 100% of isolates in a single REPC [26], to the heightened diversity noted in our study and that of Sargeant et al. [14]. Similar to the present study, Childs et al. [27] found only occasional commonality among PFGE profiles of *E. coli* O157:H7 isolates on-farm collected from hides and those collected from the colon post-harvest.

Overall genetic diversity of super-shedder PS isolates across pens was high and PS isolates as a group were not more similar than *E. coli* O157:H7 isolates overall (*P* =0.77), although within the majority of pens, PS isolates had more similar PFGE profiles than all isolates from that pen (*P* < 0.01; Table 4). In contrast, all super- and low-shedder fecal isolates across pens had more similar PFGE profiles compared to all isolates (*P* < 0.001), demonstrating a greater degree of similarity in fecal as compared to PS isolates. Genetic variation among fecal grab and PS isolates collected from the same animal may reflect the contamination of hides by *E. coli* O157:H7 originating from the feedlot environment or other cattle within the pen [27]. *Escherichia coli* O157:H7 on the hide would also be subjected to additional stresses such as irradiation, elevated temperatures and desiccation, all of which have been shown to increase phylogenetic diversity of *E. coli* O157:H7 [28]. As well, if cattle are carrying multiple strains of *E. coli* O157:H7 within the gastro-intestinal tract certain strains might preferentially survive on hides due to difference in environmental fitness among isolates [29].

**Table 4:** Average group similarity of PFGE profiles by source of isolates compared by bootstrapping analyses (n = 1000) to the similarity of PFGE profiles for all isolates within a pen or across all pens

**Feedlot**	**Pen**	**SS fecal PFGE similarity (%)**	**Signif**^**y **^**of group**	**PS PFGE similarity (%)**	**Signif of group**	**LS fecal PFGE similarity (%)**	**Signif of group**
Within pen							
A	1	57.92	NS	59.94	NS	89.82	***
	2	62.18	NS	60.75	NS	72.80	***
	3	78.23	***	58.62	***	67.04	***
	4	85.92	***	68.59	***	72.70	***
	5	68.61	**	87.03	***	70.97	***
	6	60.08	NS	73.49	**	85.72	*
	7	IN^z^	IN	IN	IN	IN	IN
B	1	69.72	***	64.81	***	66.19	***
	2	73.98	***	72.13	***	86.69	***
	3	77.66	***	68.89	***	88.99	***
	4	IN	IN	IN	IN	IN	IN
Across all pens		64.80	***	61.21	NS	64.91	***

SS fecal = fecal sample from super shedder (at least 10^4^ CFU/g feces); LS = fecal sample from low shedder (< 10^4^ CFU/g feces), PS = Perineal hide swab.

^y^Signif, significance comparing group PFGE similarity with that of all isolates in a pen or across all pens, with *** = *P* < 0.001, ** = *P* < 0.01, * = *P* < 0.05, NS = *P* > 0.05.

^z^IN, insufficient number of isolates for comparison, a minimum of 2 PFGE profiles per group are required.

### Transmission of *E. coli* O157:H7 from super-shedders to pen mates

In all feedlot pens monitored, PFGE profiles of fecal isolates from low-shedders were more similar (*P* < 0.05) than those from *E. coli* O157:H7 isolates overall (Table 4) and in 3 of 6 pens with sufficient isolates for comparison, were more similar than those of super-shedder fecal isolates. Sharing of PFGE profiles between low- and super-shedder isolates collected in the same pen was uncommon and 77% of super-shedder isolates were distinct from low-shedders within the same pen (Table 2). For 4/11 pens, PFGE profiles of all low-shedder isolates were distinct from both fecal and PS isolates of super-shedders, while 4/11 pens shared PFGE profiles of low- and super-shedder isolates only in REPC A.

Pen 2 of feedlot B had both the highest number of super-shedders detected (n = 44) and the highest number of steers positive for *E. coli* O157:H7 (n = 200). Thirty-four of the super-shedders had fecal isolates in REPCs unique to super-shedders with the remaining super-shedder isolates belonging to REPC A which colonized cattle regardless of shedding status. Similarly, pen 2 from feedlot A had a total of 36 super shedders, but 24 super-shedder fecal isolates belonged to REPCs unique to super- shedders and the remaining 12 belonged to REPC A. Pen 1 of feedlot B was notable as 8 of 9 super-shedders detected in the second sampling were housed in this pen (Table 1), but only 4/27 fecal isolates from super-shedders belonged to a REPC which also contained low-shedders (Table 2). In contrast to previously described pens, Pen 3 in feedlot A showed closer relationships among super-shedder and low-shedder PFGE profiles, as 11/12 super-shedder fecal isolates were in REPCs which also contained 46% of low-shedder fecal isolates.

In all pens of cattle with > 5 super-shedders, REPC exclusive to super-shedders were noted. In contrast, super- and low-shedders did not share PFGE subtypes of *E. coli* O157:H7 in all pens of cattle evaluated. As multiple super-shedders shared PFGE subtypes, it is possible that transmission of these subtypes from an initial super-shedder resulted in a gradual conversion of pen mates to super-shedding status. In pens where super- and low-shedders shared PFGE subtypes, it is possible that super-shedders were transmitting these subtypes to pen-mates. Due to the limited sharing of PFGE subtypes by super- and low-shedders, these results would agree with those of Dodd et al. [13] where high and low-shedding cattle in truck-loads at slaughter shared identical PFGE subtypes in feces less than 25% of the time. The high degree of similarity in PFGE subtypes of super- and low-shedder *E. coli* O157:H7 isolates reported by Cobbold et al. [8] is likely a reflection of less diversity of *E. coli* O157:H7 in pens of 8 animals compared to that noted in the commercial feedlot pens of the present study.

If high-level shedding is transient in accord with Robinson et al. [6], certain REPC might be specific to super-shedders with low-shedders in these REPC either former or future super-shedders. Conversely, as hide contamination is of crucial importance in transmission of *E. coli* O157:H7 among pen mates [9,30], subtypes of *E. coli* O157:H7 carried on hides of super-shedders might be more critical for dissemination of the organism within a feedlot pen than those within feces. Accordingly, in our previous study and source of isolates for the current study [3], presence of super-shedders within a feedlot pen increased incidence of contamination of PS with *E. coli* O157:H7, although incidence or level of fecal shedding within pens was not uniformly impacted. Analysis of PFGE subtypes of PS from low-shedders would have helped to clarify the role of super-shedders in transmission of *E. coli* O157:H7 in commercial feedlot pens, but unfortuntately was not possible in the present study due to budget restraints. Transmission of *E. coli* O157:H7 within commercial feedlot pens is undoubtedly complex and additional study will be required to confirm the relationship between fecal and hide contamination and if specific REPC are associated with super-shedders.

### Anti-microbial resistance and PFGE profiles of *E. coli* O157:H7

Anti-microbial resistance of *E. coli* O157:H7 isolates was low (3.7%; Table 5) and did not differ in frequency among low- and super-shedders (*P* =0.69; Table 6). The 3 super-shedder isolates with AMR all showed multi-drug resistance in contrast to the low-shedders where resistance to tetracycline predominated (10/14 resistant isolates). Qualitatively, no relationships among AMR and PFGE profiles were evident as resistant isolates were spread across REPC and unique isolates (data not shown).

**Table 5:** **Numbers and profiles of antibiotic resistance (AMR) of *E. coli *O157:H7 isolated from feces collected in Alberta, Canada in 2007 and number of isolates susceptible to all antibiotics (SUS) by feedlot and pen for super-shedders (SS)**^**x **^**and low-shedders (LS)**^**y **^**of *E. coli *O157:H7**

**Feedlot**	**Pen**	**No. of resistant SS isolates (%)**	**Resistance to**^**z**^	**No. of resistant LS isolates (%)**	**Resistance to**	**Total No. SUS**
A	1	0 (0)	ND	2 (12)	tetracycline, sulfasoxazole, neomycin, streptomycin , tetracycline	19
	2	1 (3)	ampicillin, sulfasoxazole, streptomycin, tetracycline	2 (6)	tetracycline, sulfasoxazole, streptomycin, sulfamethoxazole/trimethroprim	71
	3	0 (0)	ND	1(4)	tetracycline	35
	4	2 (11)	ceftazidime, streptomycin, sulfasoxazole, sulfamethoxazole/trimethro-prim	2(9)	tetracycline, tetracycline, streptomycin	41
	5	0 (0)	ND	1 (3)	sulfasoxazole, streptomycin, tetracycline	46
	6	0 (0)	ND	1(3)	tetracycline	34
	7	0 (0)	ND	0 (0)	ND	1
B	1	0 (0)	ND	3 (6)	tetracycline	78
	2	0 (0)	ND	0 (0)	ND	94
	3	0 (0)	ND	1(6)	tetracycline	26
	4	0 (0)	ND	1(9)	tetracyline	12
Total		3 (2)		14 (5)		457

^x^Super-shedder, shedding at least 10^4^ CFU E. coli O157:H7/g feces.

^y^Low-shedder, shedding < 10^4^ CFU E. coli O157:H7/g feces.

^z^Antibiotic resistance: No resistance to enrofloxacin, amoxicillin/clauvanate or cetiofur was detected.

**Table 6:** **Relationships among super-shedder status (yes/no), sampling (1 vs 2) and resistance to any antimicrobial evaluated**^**y **^**for fecal isolates of *E. coli *O157:H7 collected in Alberta Canada in 2007 from generalized linear mixed model analysis**^**z**^

**Variable**	**Odds Ratio**	**95% C.I.**	**Significance**
Sampling	1.51	0.48 – 4.78	*P* > 0.48
Super-shedder	0.94	0.31 – 2.91	*P* > 0.69

^y^Resistance detected for 17/474 isolates for antimicrobials including ampicillin, neomycin, streptomycin, sulfasoxazole, sulfamethoxazole/trimethroprim and tetracycline.

^z^Pen as random effect, covariance parameter estimate = 1.82, with standard error = 2.16.

Resistance in the current study was lower than that reported for *E. coli* O157 by Rao et al. [31] in a survey of 21 Alberta feedlots, although those authors found higher levels of AMR in newly arrived cattle during the spring as compared to pre-slaughter. In accord with Alexander et al. [32], the most common AMR in the present study was to tetracycline. Antimicrobial resistance in the current study would be consistent with that for 93 *E. coli* O157:H7 isolates previously collected from feedlot B [33] where only 3 isolates exhibited AMR to either tetracycline or chloramphenicol, and one isolate demonstrated multi-drug resistance to tetracycline, amoxicillin/clavulanic acid and ampicillin. Reasons for the low level of AMR in the present study are not known as antimicrobial use at feedlots A and B was similar to that of other feedlots in Alberta.

Multi-drug resistance has been linked to the presence of plasmids carrying multiple resistance determinants [34] and the presence of these plasmids has been demonstrated to confer fitness to environmental challenges such as acid tolerance and nutrient scarcity along with resistance to antimicrobials [35]. Accordingly, enterotoxigenic strains of *E. coli* have an increased prevalence of plasmid-mediated genes for antimicrobial resistance [36], although a relationship between virulence and AMR in *E. coli* O157:H7 has not been fully established. That all fecal isolates from super-shedders of *E. coli* O157:H7 with AMR demonstrated multi-drug resistance is intriguing, but confirming a relationship between presence of multiple drug resistance and heightened colonization with or shedding of *E. coli* O157:H7 would require additional study at locations with a higher incidence of AMR.

## Conclusions

Results of this study suggest that feedlot cattle do not remain super-shedders for extended periods, as only 1/162 steers was a super-shedder in 2 sampling periods that were at least 6 weeks apart. Approximately 30% of low-shedders in sampling 2 were super-shedders in sampling 1, indicating that high-level shedding may be related to increased persistence of *E. coli* O157:H7 in the gastro-intestinal tract. Accordingly, 15.4% of low-shedders shed *E. coli* O157:H7 in both sampling periods compared to 33.5% of super-shedders (data not shown). Based on PFGE analyses, diversity of hide and fecal isolates from individual super-shedders was high as only 2/162 super-shedder steers had hide and fecal isolates in the same REPC. The most common REPC, REPC A, was not related to shedding status (super- or low-shedder) of cattle and was also the most common REPC in both samplings, possibly reflecting increased fitness of this subtype of *E. coli* O157:H7. Overall, 77% of super-shedder isolates (fecal and PS) were distinct from low-shedder fecal isolates in the same pen. If isolates belonging to REPC A were excluded, < 10% of super-shedders shared PFGE profiles with low-shedders in the same pen. Consequently, transmission of *E. coli* O157:H7 from super-shedders to low-shedder pen mates may be limited.

## Methods

### Collection of samples

Isolates were those collected in 2007 in the study of Stephens et al. [3] (excluding samples from 1 pen lost to a freezer malfunction) and were obtained from a total of 11 pens in two commercial feedlots, with an average of 181 steers per pen (Table 1). The feedlots were both located in southern Alberta and were separated by a distance of 62 km. Steers were sampled twice: at entry to the feedlot during the months of May through October, 2007 and prior to shipment to slaughter during the months of September through December, 2007.

The animal care committee at the Lethbridge Research Centre did not evaluate studies done under commercial conditions until after publication in 2009 of revised Canadian Council of Animal Care guidelines on the the care and use of farm animals in research, teaching and testing. Therefore, this study was exempt from requiring ethical approval. However, anything more than a minimally invasive study would not have been acceptable to our commercial collaborators.

Fecal grab and perineal hide swab (100 cm^2^ area around the anus in the center of the perineum) samples were simultaneously obtained from each steer. Fecal samples were obtained by rectal palpation using a clean glove for each animal. Feces were placed in Whirl-Pak® bags and transported to the laboratory on ice. Perineal swab samples (PS) were obtained using a sterile SpongeSicle® hydrated with 25 mL of PBS with a new SpongeSicle® used for each animal. Each SpongeSicle® was placed in a separate Whirl-Pak® bag along with 45 mL of modified *E. coli* broth with 20 mg/L novobiocin (mEC-nov) and transported to the laboratory at ambient temperature. All samples were delivered to the laboratory for analysis within a period of 12 h and refrigerated at 5°C until completion of *E. coli* O157:H7 detection and enumeration.

### *Escherichia coli* O157:H7 Detection

Bags of feces were manually blended prior to sub-sampling and 1 g of feces was added to 9 mL of mEC-nov and incubated at 37°C for 6 h. Perineal swab samples were incubated in the original transport media for 18 h at 37°C. After enrichment, a 1 mL aliquot of each sample type (fecal grab and PS) was subjected to immunomagnetic separation (IMS) using Dynabeads anti-O157 and a PickPen® magnetic particle separation device as per manufacturers’ instructions. Fifty-μL of the bead-bacteria mixture was plated onto sorbitol MacConkey agar supplemented with 2.5 mg/L potassium tellurite and 0.05 mg/L cefixime (CT-SMAC) and plates were incubated for 16 h at 37°C. Up to 3 sorbitol negative (clear) colonies per plate were subjected to agglutination using an *E. coli* O157 latex kit. One isolate per sample was further subjected to multiplex PCR assays for the detection of the *stx*1, *stx*2, *eaeA,* and *flicC* (H7) genes and isolates with *eaeA, flicC* and either or both of *stx*1 and *stx*2 were confirmed as *E. coli* O157:H7 [37].

### *Escherichia coli* O157 Enumeration

Fecal grab samples that were positive for *E. coli* O157 were serially diluted (1 g of feces in 9 mL of mEC-nov) and 100 μL of the 10^-2^ and 10^-3^ dilutions were plated in duplicate onto CT-SMAC. The CT-SMAC plates were incubated for 16 h at 37°C. Up to 5 sorbitol-negative (clear) colonies per plate were subjected to agglutination using an *E. coli* O157 latex kit. Sorbitol-negative colonies were counted on each duplicate plate, dilution calculations were performed, adjustments for the proportion of positive agglutinations out of 5 were made, and counts were recorded in CFU/g. Super-shedders were defined as cattle that had at least 10^4^ CFU *E. coli* O157:H7/g feces, while low-shedders had < 10^4^ CFU/g feces.

### Pulsed-field gel electrophoresis

Isolates confirmed as *E. coli* O157:H7 from fecal grabs of super-shedders (n = 162), PS of super-shedders (n = 137) and fecal grabs of low-shedder pen mates (n = 496) were sub-typed by PFGE using *Xba*I restriction according to the standard 1-d protocol [38]. Isolates from low-shedder PS (n = 1023) were collected but excluded from analysis due to time and labor constraints. One isolate of *E. coli* O157:H7 from each positive sample was typed by PFGE using a CHEF DR II electrophoresis unit (Bio-Rad Laboratories, Mississauga, ON, Canada). Banding patterns were viewed with UV illumination and photographed using the Speedlight Platinum Gel Documentation System (Bio-Rad, Mississauga, ON).

### Antimicrobial resistance testing

Isolates (n = 474) were tested for resistance to 11 antimicrobials using anti-microbial disk susceptibility tests [39]. Eleven antibiotic discs were applied using a 12-place BBL Sensi-Disc™ disc dispenser (VWR, Intl., Edmonton, AB.) The antimicrobials tested included enrofloxacin (5 μg), streptomycin (10 μg), amoxicillin/clavulanate (20/10 μg), ceftiofur (30 μg), ampicillin (10 μg), sulphmethoxazole/trimethoprim (23.75/1.25 μg), ceftazadime (30 μg), oxytetracycline (30 μg), neomycin (30 μg), florfenicol (30 μg) and sulfasoxazole (0.25 μg). *Escherichia coli* ATCC strain 25922 and *Enterococcus faecalis* ATCC 29212 were used as controls in accordance with Clinical Laboratory Standards Institute Guidelines [39]. Zone diameters were measured after 18 h incubation at 37^o^ C using a BIOMIC® V3 digital imaging system and software (Giles Scientific Inc., Santa Barbara, CA).

### Statistical analyses

Pulse-field gel electrophoresis patterns in the digital images were classed as unique or grouped into restriction endonuclease digestion pattern clusters (REPC; 90% or greater similarity) using Dice similarity coefficients, unweighted pair group methods arithmetic average algorithm, 1% position tolerance and 0.5% optimization (BioNumerics 6.5, Applied Maths BVBA, Sin-Martens-Latem, Belgium). Within- and between-group similarities of PFGE profiles for super- and low-shedder isolates within and across feedlot pens were tested using the Dimensioning Techniques package of BioNumerics. For these analyses, binary character profiles using the band-matching option were created after clustering using Dice coefficients. Group similarity was then evaluated using bootstrapping analyses (n = 1000), with significant differences reported at *P* < 0.05. Within a pen, similarity of a group (super-shedder fecal isolates, super-shedder perineal swab, or low-shedder fecal isolates) was compared to that of random samples from all isolates in the pen as part of the bootstrapping analysis to determine if similarity among samples in a group was greater than that expected by chance. Membership in REPC A as compared to all other REPC and data generated from antimicrobial resistance profiles of super- and low-shedder fecal isolates were compared using a logit link function and binomial distribution within the GLIMMIX procedure of SAS (SAS 9.1; Cary, NC, USA), with shedding status (super- or low-shedder) and sampling [1,2] as the independent variables, and feedlot pen modeled as a random intercept, with signficiant differences at *P* < 0.05 and REML used to estimate the variance component.

## Competing interests

The authors declare that they have no competing interests.

## Authors’ contributions

CA analysed data using the BioNumerics program. KS performed other statistical analyses, participated in study design and drafted the manuscript. TM contributed to study design and critically reviewed the drafted manuscript. All authors read and approved the final manuscript.

## Supplementary Material

Additional file 1 **Figure 1.** Dendrogram of restriction endonuclease clusters (REPC) from sampling 1, showing REPC shared with sampling 2 (A through E). REPC exlusive to sampling 1 are not labeled. Click here for file

Additional file 2 **Figure 2.** Dendrogram of restriction endonuclease clusters (REPC) from sampling 2, showing REPC shared with sampling 1 (A through E). Click here for file

## References

[B1] Matthews L McKendrick IJ Ternet H Gunn GJ Synge B Woolhouse MEJ Super-shedding cattle and the transmission dynamics of Escherichia coli O157 Epidemiol Infect 2006 134 131 142 16409660 10.1017/S0950268805004590 PMC2870353

[B2] Carlson BA Nightingale KK Mason GL Ruby JR Choat WT Loneragan GH Smith GC Sofos JN Belk KE Escherichia coli O157:H7 strains that persist in feedlot cattle are genetically related and demonstrate and enhanced ability to adhere to intestinal epithelial cells Appl Environ Microbiol 2009 75 5927 5937 10.1128/AEM.00972-09 19617387 PMC2747851

[B3] Stephens TP McAllister TA Stanford K Perineal swabs reveal the effect of super shedders on the transmission of Escherichia coli O157H7 in commercial feedlots J Anim Sci 2009 87 4151 4160 10.2527/jas.2009-1967 19684276

[B4] Chase-Topping M Gall D Low C Matthews L Woolhouse M Super-shedding and the link between human infection and livestock carriage of Escherichia coli O157 Nat Rev Micro 2008 6 904 912 10.1038/nrmicro2029 PMC5844465 19008890

[B5] Omisakin F MacRae M Odgen ID Strachan NJC Concentration and prevalence of Escherichia coli O157 in cattle feces at slaughter Appl Environ Microbiol 2003 69 2444 2447 10.1128/AEM.69.5.2444-2447.2003 12732509 PMC154535

[B6] Robinson SE Brown PE Wright EJ Hart CA French NP Quantifying within- and between-animal variation and uncertainty associated with counts of Escherichia coli O157 occurring in naturally infected cattle faeces J R Soc Interface 2009 6 169 177 10.1098/rsif.2008.0183 18647739 PMC2658787

[B7] Ferens WA Hovde CJ Escherichia coli O157:H7: animal reservoir and sources of human infection Foodborne Path Dis 2011 8 465 487 10.1089/fpd.2010.0673 PMC3123879 21117940

[B8] Cobbold RN Hancock DD Rice DH Berg J Stilborn R Hovde CJ Besser TE Rectoanal junction colonization of feedlot cattle by Escherichia coli O157:H7 and its association with supershedders and excretion dynamics Appl Environ Microbiol 2007 73 1563 1568 10.1128/AEM.01742-06 17220263 PMC1828767

[B9] Stanford K Stephens TP McAllister TA Use of model super-shedders to define the role of pen floor and hide contamination in the transmission of Escherichia coli O157:H7 J Anim Sci 2011 89 237 244 10.2527/jas.2010-3088 20852081

[B10] Fox JT Renter DG Sanderson MW Nutsch AL Shi X Nagaraja TG Associations between the presence and magnitude of Escherichia coli O157 in feces at harvest and contamination of pre-intervention beef carcasses J Food Protect 2008 71 1761 1767 10.4315/0362-028x-71.9.1761 18810859

[B11] Jacob ME Renter DG Nagaraja TG Animal and truckload-level associations between Escherichia coli O157:H7 in feces and on hides at harvest and contamination of pre-evisceration beef carcasses J Food Protect 2010 73 1030 1037 10.4315/0362-028x-73.6.1030 20537257

[B12] Avery SM Lievana E Hutchinson ML Buncic S Pulsed-field gel electrophoresis of related Escherichia coli O157 isolates associated with beef cattle and comparison with unrelated isolates from animals, meats and humans Int J Food Microbiol 2004 92 161 169 10.1016/j.ijfoodmicro.2003.09.007 15109793

[B13] Dodd CC Renter DG Fox JT Shi X Sanderson MW Nagaraja TG Genetic relatedness of Escherichia coli O157 isolates from cattle feces and pre-intervention beef carcasses Foodborne Path Dis 2010 7 357 365 10.1089/fpd.2009.0415 19911900

[B14] Sargeant JM Shi X Sanderson MW Renter DG Nagaraja TG Pulsed-field gel electrophoresis patterns of Escherichia coli O157 isolates from Kansas feedlots Foodborne Path Dis 2006 3 251 258 10.1089/fpd.2006.3.251 16972773

[B15] Lefebvre B Diarra MS Giguere K Roy G Michaud S Malouin F Antibiotic resistance and hypermutability of Escherichia coli from feedlot cattle treated with growth-promoting agents J Food Protect 2005 68 2411 2419 10.4315/0362-028x-68.11.2411 16300081

[B16] Van Donkersgoed J Berg J Potter A Hancock D Besser T Rice D LeJeune J Klashinsky S Environmental sources and transmission of Escherichia coli O157 in feed lot cattle Can Vet J 2001 42 714 720 11565371 PMC1476616

[B17] Stanford K Bach SJ Marx TH Jones S Hansen JR Wallins GL Zahiroddini H McAllister TA Monitoring Escherichia coli O157:H7 in inoculated and naturally infected feedlot cattle and their environment J Food Protect 2005 68 26 33 10.4315/0362-028x-68.1.26 15690800

[B18] Matthews L Low JC Gally DL Pearce MC Mellor DJ Heesterbeek JAP Chase-Topping M Naylor SW Shaw DJ Reid J Woolhouse MEJ Heterogenous shedding of Escherichia coli O157 in cattle and its implications for control Proc Natl Acad Sci 2006 103 547 552 10.1073/pnas.0503776103 16407143 PMC1325964

[B19] Tenover FC Arbeit RD Goering RV Mickelsen PA Murray BE Pershing DH Swaminathan B Interpreting chromosomal DNA restriction patterns produced by pulsed-field gel electrophoresis: criteria for bacterial strain typing J Clin Microbiol 1995 33 2233 2239 7494007 10.1128/jcm.33.9.2233-2239.1995 PMC228385

[B20] Vali L Hamouda A Pearce MC Knight HI Evans J Amyes SGB Detection of genetic diversity by pulsed-field gel electrophoresis among Escherichia coli O157 isolated from bovine faecal samples by immunomagnetic separation technique Lett Appl Microbiol 2007 44 19 23 10.1111/j.1472-765X.2006.02034.x 17209809

[B21] Brun E Holstad G Kruse H Jarp H Within-sample and between-sample variation of antimicrobial resistance in fecal Escherichia coli isolates from pigs Microb Drug Resist 2002 8 385 391 10.1089/10766290260469660 12523637

[B22] Vali L Wisely KA Pearce MC Turner EJ Knight HI Smith AW Amyes SGB High-level genotypic variation and antibiotic sensitivity among Escherichia coli O157 strains isolated from two Scottish beef cattle farms Appl Environ Microbiol 2004 70 5947 5954 10.1128/AEM.70.10.5947-5954.2004 15466537 PMC522067

[B23] Prendergast DM Lendrum L Pearce R Ball C McLernon J O’Grady D Scott L Fanning S Egan J Guitierrez M Verocytotoxigenic Escherichia coli O157 in beef and sheep abattoirs in Ireland and characterisation of isolates by pulsed-field gel electrophoresis and multi-locus variable number of tandem repeat analysis Int J Food Microbiol 2011 144 519 527 10.1016/j.ijfoodmicro.2010.11.012 21115208

[B24] Rice DH McMenamin KM Prichett LC Hancock DD Besser TE Genetic subtyping of Escherichia coli O157 isolates from 41 pacific northwest USA cattle farms Epidemiol Infect 1999 122 479 484 10.1017/S0950268899002496 10459653 PMC2809644

[B25] Arthur TA Bosilevac JM Britchta-Harhay DM Guerin MN Kalchayanand N Shackelford SD Wheeler TL Koohmaraie M Transportation and lairage environment effects on prevalence, numbers and diversity of Escherichia coli O157:H7 on hides and carcasses of beef cattle at processing J Food Protect 2007 70 280 286 10.4315/0362-028x-70.2.280 17340859

[B26] Stanford K Croy D Bach SJ Wallins GL Zahiroddini H McAllister TA Ecology of Escherichia coli O157:H7 in commercial dairies in southern Alberta J Dairy Sci 2005 88 4441 4451 10.3168/jds.S0022-0302(05)73131-3 16291636

[B27] Childs KD Simpson CA Warren-Serna W Bellenger G Centrella B Bowling RA Ruby J Stefanek J Vote DJ Choat T Scanga JA Sofos JN Smith GC Belk KE Molecular characterization of Escherichia coli O157:H7 hide contamination routes: feedlot to harvest J Food Protect 2006 69 1240 1247 10.4315/0362-028x-69.6.1240 16786841

[B28] Cooley MB Carychao D Nguyen K Whitehand L Mandrell R Effects of environmental stress on stability of tandem repeats in Escherichia coli O157:H7 Appl Environ Microbiol 2010 76 3398 3400 10.1128/AEM.02373-09 20348301 PMC2869130

[B29] Avery SM Buncic S Escherichia coli O157 diversity with respect to survival during drying on concrete J Food Protect 2003 66 780 786 10.4315/0362-028x-66.5.780 12747685

[B30] McGee P Scott L Sheridan JJ Earley B Leonard N Horizontal transmission of Escherichia coli O157:H7 during cattle housing J Food Prot 2004 67 2651 2656 15633666 10.4315/0362-028x-67.12.2651

[B31] Rao S Van Donkersgoed J Bohaychuk V Besser T Song X Wagner B Hancock D Renter D Dargatz D Morley PS Antimicrobial drug use and antimicrobial resistance in enteric bacteria among cattle from Alberta feedlots Foodborne Path Dis 2010 7 449 457 10.1089/fpd.2009.0400 19958100

[B32] Alexander TW Inglis GD Yanke LJ Topp E Read RR Reuter T McAllister TA Farm-to-fork characterization of Escherichia coli associated with feedlot cattle with a known history of antimicrobial use Int J Food Microbiol 2010 137 40 48 10.1016/j.ijfoodmicro.2009.11.008 19963297

[B33] Aslam M Stanford K McAllister TA Characterization of antimicrobial resistance and seasonal prevalence of Escherichia coli O157:H7 recovered from commercial feedlots in Alberta Canada Letts Appl Micro 2010 50 320 326 10.1111/j.1472-765X.2010.02798.x 20102509

[B34] Sherley M Gordon DM Collignon PJ Evolution of multi-resistance plasmids in Australian clinical isolates of Escherichia coli Microbiol 2004 150 1539 1546 10.1099/mic.0.26773-0 15133115

[B35] Singer RS Ward MP Maldonado G Can landscape ecology untangle the complexity of antibiotic resistance? Nat Rev Microbiol 2006 4 943 952 10.1038/nrmicro1553 17109031

[B36] Smith MG Jordan D Chapman TA Chin JJC Barton MD Do TN Fahy VA Fairbrother JM Trott DJ Antimicrobial resistance and virulence gene profiles in multi-drug resistant enterotoxigenic Escherichia coli isolated from pigs with post-weaning diarrhoea Vet Microbiol 2010 145 299 307 10.1016/j.vetmic.2010.04.004 20688440

[B37] Gannon VP D’Souza S Graham T King RK Specific identification of Escherichia coli O157:H7 using multiplex PCR assay Adv Exp Med Biol 1997 412 81 82 9191994 10.1007/978-1-4899-1828-4_10

[B38] Ribot EM Fair MA Gautom R Cameron DN Hunter SB Swaminathan B Barrett TJ Standardization of pulsed-field gel electrophoresis protocols for the subtyping of Escherichia coli O157:H7, Salmonella and Shigella for PulseNet Foodborne Path Dis 2006 3 59 66 Available online: http://www.cdc.gov/pulsenet/protocols.htm. Accessed June 24, 2011 10.1089/fpd.2006.3.59 16602980

[B39] Clinical and Laboratory Standards Institute Performance standards for antimicrobial disk susceptibility tests; approved standards – Ninth ed 2006

