# Supplementary figures and images for: Genetic diversity and antimicrobial resistance among isolates of Escherichia coli O157: H7 from feces and hides of super-shedders and low-shedding pen-mates in two commercial beef feedlots
Source: BMC Vet Res. 2012 Sep 26;8:178. doi: 10.1186/1746-6148-8-178 (PMC3582550; doi:10.1186/1746-6148-8-178)

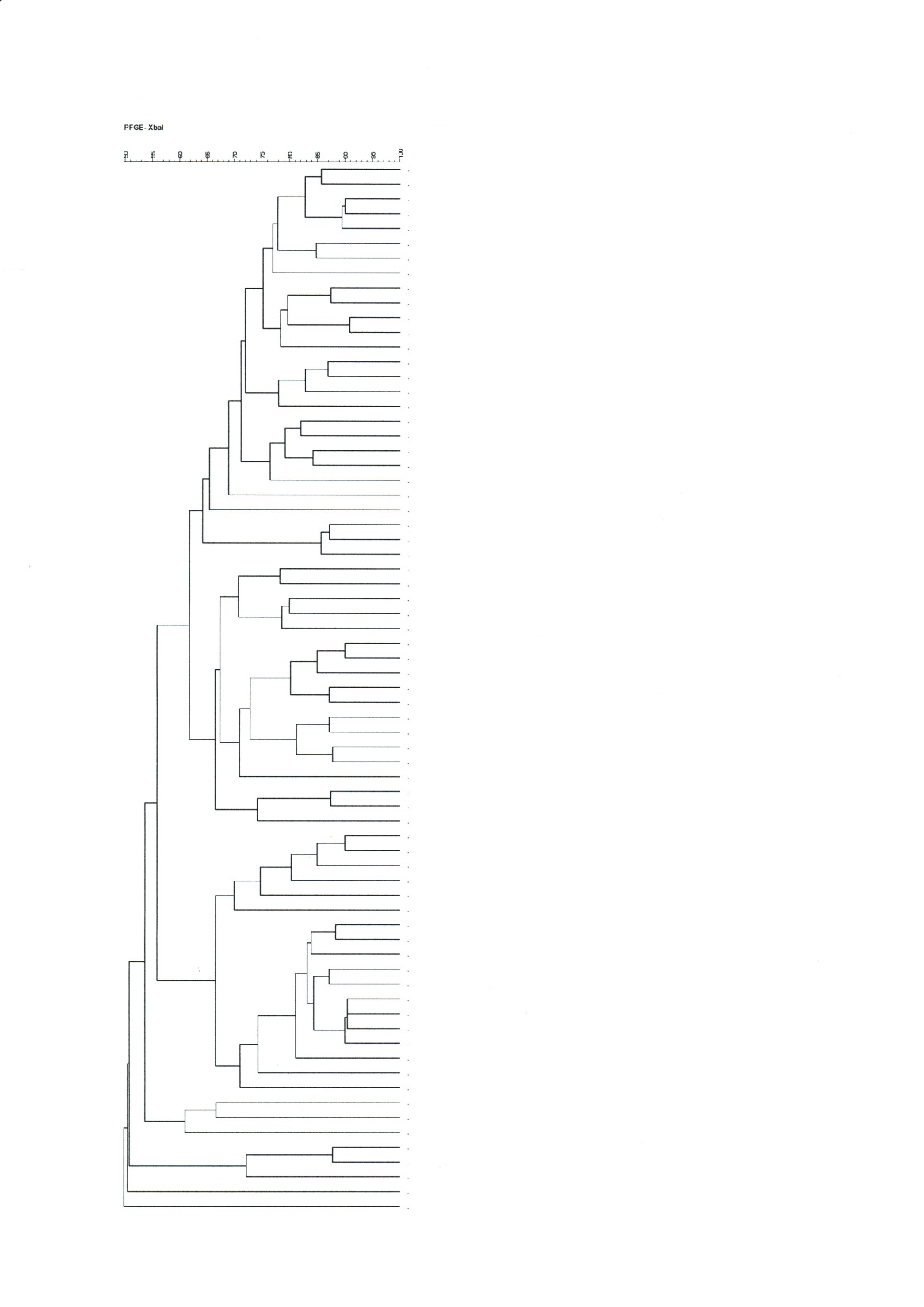

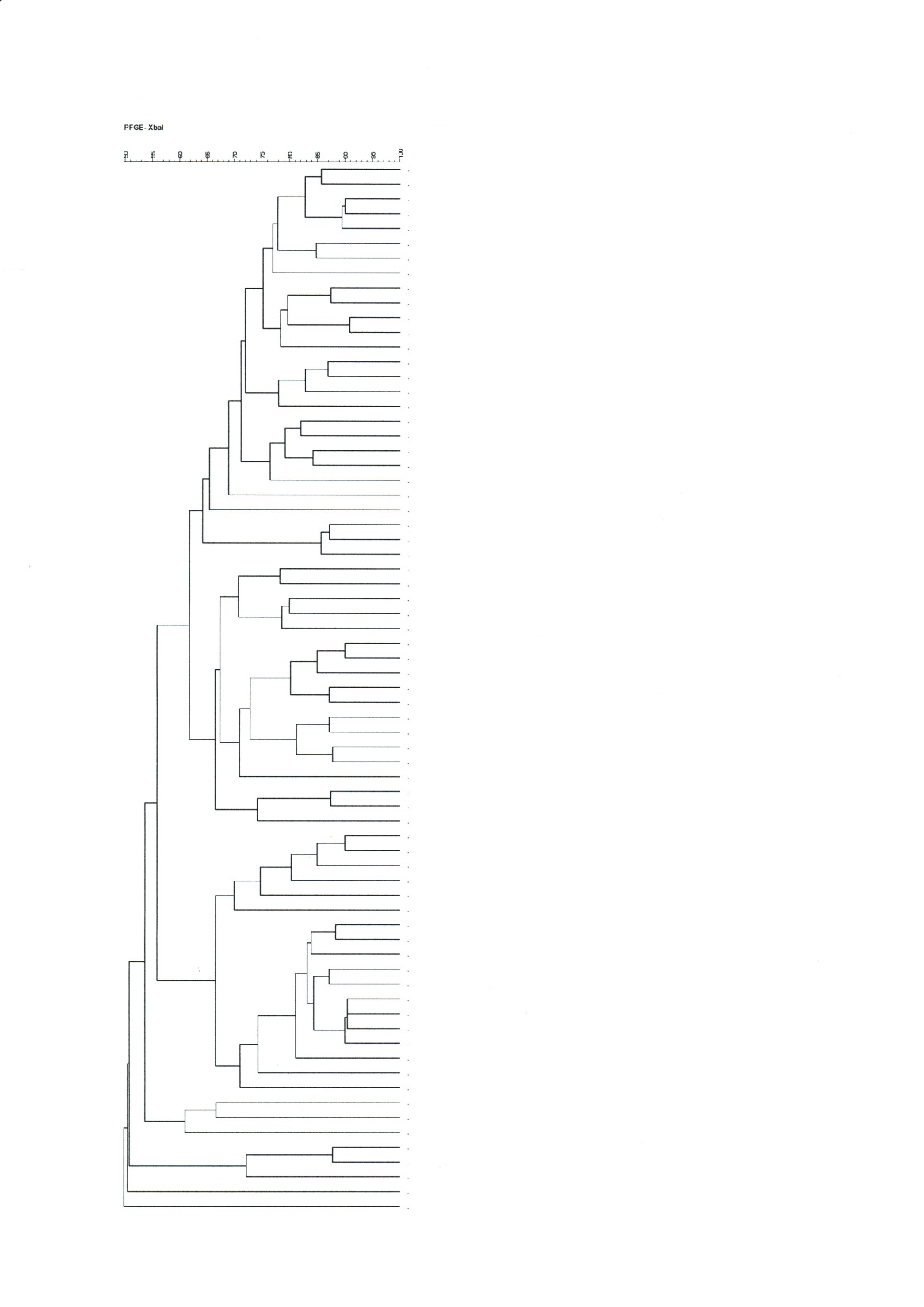


Stanford et al. Fig 1.

REPC A

REPC B

REPC C

REPC D

REPC E

Supplement: Additional file 1 — Figure 1. Dendrogram of restriction endonuclease clusters (REPC) from sampling 1, showing REPC shared with sampling 2 (A through E). REPC exlusive to sampling 1 are not labeled. [file 1746-6148-8-178-S1.docx]
