# Supplementary material for: Genetic diversity and antimicrobial resistance among isolates of Escherichia coli O157: H7 from feces and hides of super-shedders and low-shedding pen-mates in two commercial beef feedlots
Source: BMC Vet Res. 2012 Sep 26;8:178. doi: 10.1186/1746-6148-8-178 (PMC3582550; doi:10.1186/1746-6148-8-178)
Supplement: Additional file 2 — Figure 2. Dendrogram of restriction endonuclease clusters (REPC) from sampling 2, showing REPC shared with sampling 1 (A through E). [file 1746-6148-8-178-S2.docx]

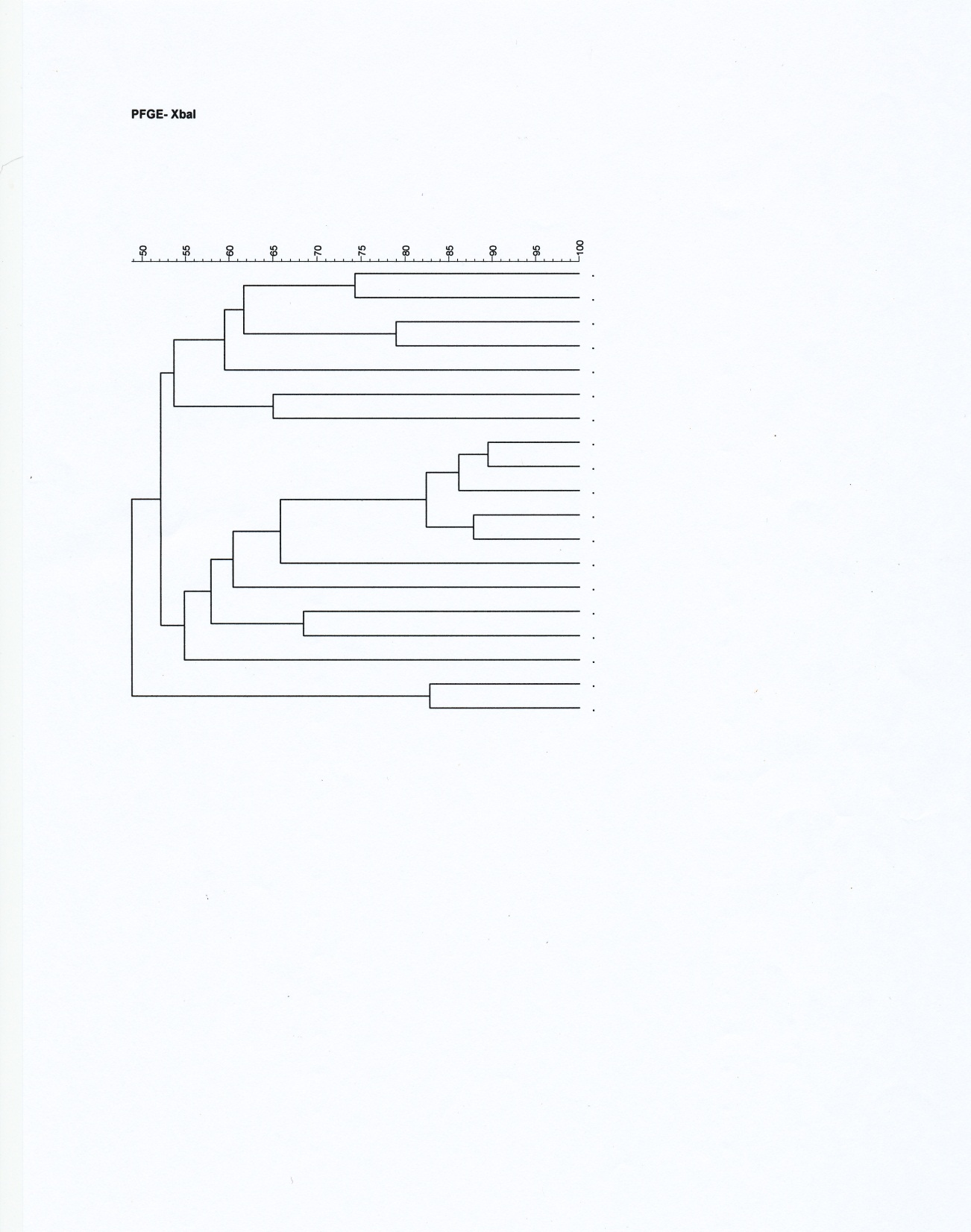


Stanford et al. Fig 2.

Sampling 2 only Sampling 2 only

Sampling 2 only

Sampling 2 only

Sampling 2 only

Sampling 2 only

Sampling 2 only

Sampling 2 only

Sampling 2 only

REPC A

REPC B

REPC C

Sampling 2 only

REPC D

Sampling 2 only

Sampling 2 only

Sampling 2 only

REPC E

Sampling 2 only

Sampling 2 only

Sampling 2 only
